# Effect of CXCL12/CXCR4 on increasing the metastatic potential of non-small cell lung cancer *in vitro* is inhibited through the downregulation of CXCR4 chemokine receptor expression

**DOI:** 10.3892/ol.2014.1837

**Published:** 2014-01-29

**Authors:** SONGPING XIE, WENHUI ZENG, GUOHUA FAN, JIE HUANG, GANJUN KANG, QING GENG, BANGCHANG CHENG, WEI WANG, PING DONG

**Affiliations:** Department of Thoracic Surgery, Renmin Hospital of Wuhan University, Wuhan, Hubei 430060, P.R. China

**Keywords:** chemokine, CXCR4, CXCL12, RNA interference, non-small cell lung cancer

## Abstract

Lung cancer ranks as the most common type of cancer in males worldwide. Although great advances have been achieved in chemotherapy and radiotherapy, the long-term survival rate of lung cancer patients has not improved significantly. Dissemination of lung cancer in the thoracic cavity and metastatic spread to the liver, bone and brain are characteristic of non-small cell lung cancer (NSCLC), constituting the primary source of morbidity and mortality in lung cancer. Increasing evidence also indicates that the CXC chemokine receptor 4 (CXCR4)/chemokine CXC motif ligand 12 (CXCL12) chemokine axis is important for the cell invasion and migration of lung cancer. CXCR4 is a G protein-coupled receptor with a major role in lymphocyte homing. Its ligand, CXCL12, is secreted by target organs and functions as a highly efficient chemotactic factor for T cells, monocytes, pre-B cells, dendritic cells and myeloid bone marrow-derived cells. In the current study, recombinant CXCR4-specific small interfering RNA-pBSilence1.1 plasmids were constructed and transfected into the A549 NSCLC cell line *in vitro*. Reverse transcription polymerase chain reaction and western blotting revealed that CXCR4 was downregulated in transfected cells compared with control cells. The results of MTT and Transwell migration assays indicated that the specific downregulation of CXCR4 inhibited cell growth, invasiveness and migration. Thus, siRNA targeting of CXCR4 may effectively inhibit the effect of CXCL12/CXCR4 on increasing the metastatic potential of NSCLC.

## Introduction

Lung cancer ranks as the most frequent type of cancer in males worldwide with increasing incidence rates according to the epidemiology data (1). Although great advances have been achieved in chemotherapy and radiotherapy, only small, incremental improvements in the outcome of lung cancer have been realized, and the long-term survival rate of lung cancer patients has not improved significantly. Metastatic spread to the regional lymph nodes, liver, bone and brain, which is characteristic of non-small cell lung cancer (NSCLC), constitutes the primary source of morbidity and mortality in lung cancer. The majority of patients present with locally advanced (37%) or metastatic (38%) disease at the time of diagnosis (2,3). Increased understanding of the molecular mechanisms and processes underlying the metastasis of lung cancer cells is critical for developing effective new therapies for lung cancer. Specific receptors have been identified that are required for cancer cells to proliferate and migrate, modulating cancer progression (4). Thus, receptors may represent opportunistic targets for engineering vehicles that localize in primary and distal lung tumors.

Chemokines are a superfamily of chemoattractant, cytokine-like proteins that bind to and activate a family of chemokine receptors. Over 50 chemokines have been identified and can be divided into four families (CXC, CX3C, CC and C), according to the positions of four conserved cysteine residues (5). Chemokines, which are structurally and functionally similar to growth factors, bind to G protein-coupled receptors on leukocytes and stem cells and process guanine nucleotide-binding proteins to initiate intracellular signaling cascades that promote migration towards the chemokine source (6). Chemokine CXC motif ligand 12 (CXCL12) is a CXC chemokine that interacts with a specific receptor, CXC chemokine receptor 4 (CXCR4). There is increasing evidence to suggest that the CXCL12/CXCR4 axis functions as a critical molecular determinant for events, including maintaining embryo development, mediating immune and inflammatory reactions and the modulation of the hematopoietic system, involving HIV infection and angiogenesis (7). CXCR4 has previously been highlighted for its role in cancer metastasis. The CXCL12/CXCR4 axis is important for activating a plethora of phenomena, including chemotaxis, invasion, tumorigenicity and angiogenesis and proliferation in cancer, particularly in the process of organ-selective metastasis (6,8,9), demonstrating that tumor cells expressing a high level of CXCR4 exhibit metastasis to target tissues (lung, liver and bone). The target tissues express high levels of CXCL12, allowing tumor cells to directionally migrate to target organs via the CXCL12-CXCR4 chemotactic axis. CXCR4 is hypothesized to be involved in cancer invasion and metastasis, and higher levels of this receptor are associated with higher grades and poor prognosis of cancer (10,11). Notably, several retrospective studies have also examined the role of CXCR4 in NSCLC by investigating the association between CXCR4 expression with clinical outcome; NSCLC patients with greater CXCR4 expression on the surface of tumor cells have been observed to be more likely to have metastatic disease (12,13). Few studies have investigated the effects of small interfering RNA (siRNA)-directed inhibition of CXCR4 in NSCLC. To gain further insight into the effect of CXCR4 in the A549 lung cancer cell line, CXCR4 expression was selectively knocked down in the present study using RNAi. The effect of CXCL12/CXCR4 on the metastatic potential of NSCLC was also observed.

## Materials and methods

### Cell culture

The A549 human lung cancer cell line was purchased from the Archives Center of Wuhan University (Wuhan, China) and cultured in RPMI-1640 culture medium containing 10% fetal bovine serum, 100 mg/l penicillin and 100 mg/l streptomycin.

### Construction of pBSilence1.1-CXCR4-siRNA

The short hairpin RNA (shRNA) sequence with short hairpin structure was designed for the coding region sequence by online design software, BLOCK-iT™ RNAi Designer (Invitrogen Life Technologies, Carlsbad, CA, USA), according to the design principles of the RNA interference (RNAi) sequence. Sequences with nonspecific inhibition to other genes were excluded following BLAST homology analysis. The following three pairs of shRNA were designed: i) CXCR4-1-1.1 sense, 5′-CACCTGGGCAATGGATTGGTCATTTCAAGACGATGACCAATCCATTGCCCATTTTTTG-3′ and antisense, 5′-AGCTCAAAAAATGGGCAATGGATTGGTCATCGTCTTGAAATGACCAATCCATTGCCCA-3′; ii) CXCR4-2-1.1 sense, 5′-CACCGTGGCAAACTGGTACTTTGTTCAAGACGCAAAGTACCAGTTTGCCACTTTTTTG-3′ and antisense, 5′-AGCTCAAAAAAGTGGCAAACTGGTACTTTGCGTCTTGAACAAAGTACCAGTTTGCCAC-3′; and iii) CXCR4-3-1.1 sense, 5′-CACCGGCTGAAAAGGTGGTCTATTTCAAGACGATAGACCACC TTTTCAGCCTTTTTTG-3′ and antisense, 5′-AGCTCAAAAAAGGCTGAAAAGGTGGTCTATCGTCTTGAAATAGACCACCTTTTCAGCC-3′. pBSilence1.1 plasmid was completely digested by *Bsa*I, and 1% agarose gel electrophoresis was used to recycle the large fragments. Each pair of shRNA-CXCR4 provided the corresponding sequence following annealing. T4 ligase was used to associate the annealing products CXCR4-1, -2 and -3 with pBSilence1.1 linearized plasmid at 16°C overnight. The positive clone was obtained by transforming *E. coli* DH5α and extracting the plasmids. Following *Sac*I digestion, 1% agarose gel electrophoresis and sequencing identification were performed.

### Cell culture and transfection

The A549 human lung cancer cell line was cultured in RPMI-1640 culture medium containing 10% fetal bovine serum, 100 U/ml penicillin and 100 ng/ml streptomycin. The exponentially growing cells were seeded and cultured in six-well culture plates. Once the cell density had increased to 60–80%, Lipofectamine™ 2000 liposome transfection kit (Invitrogen Life Technologies) was used to transfect the plasmid into the cells according to the manufacturer’s instructions, and the transfection solution was discarded after 4–6 h. Following cultivation for two days, the original culture medium was discarded and screened by RPMI-1640 culture medium containing 400 mg/l hygromycin, and the anti-hygromycin cells were collected after two weeks.

Cells were harvested 48 h following transfection for the evaluation of CXCR4 expression. The transfection efficiency was monitored by measuring the percentage of fluorescent cells among 500 total cells using fluorescence microscopy (model 7900; ABI, Vernon, CA, USA).

### Detection of CXCR4 mRNA expression by reverse-transcription polymerase chain reaction (RT-PCR)

Following transfection for 48 h, total RNA was extracted from the lung cancer cell line A549 by TRIzol reagent and quantified by UV spectrophotometer. In addition, purity and RNA concentration were measured by a UV spectrophotometer (model 752; Shimadzu Corp., Kyoto, Japan). cDNA was obtained by reverse transcription and was used as a template for PCR amplification of the target genes and β-actin was used as standard control. The amplification program used was as follows: Denaturation for 4 min at 94°C, 30 sec at 94°C, 30 sec at 52°C, 5 sec at 72°C and, following 30 cycles, the total extension was 4 min at 72°C. The CXCR4 primers used were 5′-CCGTGGCAAACTGGTACTTT-3′ and 5′-GACGCCAACATAGACCACCT-3′ and the length of the product was 188 bp. The β-actin primers used were upstream, 5′-CACGATGGAGGGGCCGGACTCATC-3′ and downstream, 5′-TAAAGACCTCTATGCCAACACAGT-3′ (Tm=56°C). The PCR products were collected for electrophoresis with 5% agarose gel and imaging. The absorbance value of each strip was measured following analysis by a gel imaging system (Gel-Doc, Bio-Rad, Hercules, CA, USA).

### Western blot analysis of CXCR4 protein expression in A549 cells following transfection

Following transfection for 48 h, the A549 lung cancer cell line was washed with phosphate-buffered saline and 50 μl lysis buffer solution was added. Cells were collected and allowed to stand for 30 min at 4°C, and then centrifuged at 13,800 × g for 20 min. Next, the supernatant was collected and total protein concentration was measured by BCA assay (P0010; Beyotime, Shanghai, China). Protein (50 μg) was obtained for 12% polyacrylamide gel electrophoresis and electrically transferred to PVDF membrane, which had been soaked in Tris-Buffered Saline and Tween 20 (TBST) containing 5% skimmed milk powder. Nonspecific antigens were blocked for 2 h at room temperature. Mouse anti-human CXCR4 polyclonal antibody (1:400) was added and the membranes were incubated overnight at 4°C, before being washed with TBST containing 5% skimmed milk powder. Horseradish peroxidase-labeled goat anti-rabbit secondary antibody (1:50,000) was added and the mixture was reacted at room temperature for 2 h. The chemiluminescent substrate (NCI5079; Thermo Fisher Scientific, Rockford, IL, USA), ECL, was added following membrane washing, and the results were analyzed by a GIS image analysis system (Bio-Rad).

### Detection of cell proliferation by MTT assay

The stably transfected A549 lung cancer cell line was prepared for single cell suspension with RPMI-1640 medium containing 10% inactivated fetal bovine serum and the cell density was adjusted to lx10^8^/ml. Cells were added to 96-well plates at a density of 1×10^4^/100 μl. The following five groups were set up: Group A, A549 cell line plus RPMI-1640 and 10% newborn calf serum (NBS); group B, empty vector A549 cell line plus RPMI-1640 and 10% NBS; group C, A549 cell line plus RPMI-1640, 10% NBS and 100 ng/ml CXCL12; group D, A549 cell line, following RNAi, plus RPMI-1640, 10% NBS and 100 ng/ml CXCL12; and group E, A549 cell line, following RNAi, plus RPMI-1640 and 10% NBS. MTT solution (20 μl; 5 mg/ml) was added to each well after 24, 48 and 72 h, respectively, and incubated for 4 h. Following termination of the culture, the culture medium in each well was removed and discarded. Next, 150 μl DMSO was added to each well for full dissolution of the crystals. Cell proliferation was indicated by the absorption value (measured using BE2100 system, Bug lab, Concord, CA, USA) of each well at a wavelength of 570 nm.

### Detection of A549 cell migration capability by Transwell migration assay

Polycarbonate microporous membranes (pore size, 8 μm) were paved between the upper and lower Transwell chambers. Different concentrations of CXCL12 (0, 30 and 100 ng/ml, groups A, B and C, respectively) were added to the lower section of a Transwell chamber. Equal cell numbers of A549 were seeded in the upper chamber in the medium without CXCL12 (200 μl A549 cell suspension with a density of 1×105/ml). The effect of RNAi on chemotaxis migration was assessed by another Transwell-assay. Various concentrations of CXCL12 (100 and 0 ng/ml, groups D and E, respectively) were added to the lower section of a Transwell chamber. Equal cell numbers of RNA-interfered A549 were seeded in the upper chamber in medium without CXCL12 (200 μl RNA-interfered A549 cell suspension with a density of 1×10^5^/ml). Cells were cultured for 24 h in a wet incubator at 37°C with 5% CO_2_, prior to the removal of the small chamber. Cells on the membrane were removed carefully with a swab. Methanol was used to fix migration and was adhesive to the cells of the lower chamber. Next, conventional hematoxylin and eosin staining was carried out. Five fields of view (up, down, left, right and center) were selected under a light microscope (magnification, ×200; IX71, Olympus, Japan), cells in the lower chamber were counted and the mean value was representative of infiltration strength value. The cell migration inhibition ratio was calculated as follows: Cell migration inhibition ratio (%)= [number of migrating cells in the nonsilencing double-stranded (ds) RNA group - number of migrating cells in the siRNA group] / number of migrating cells in the nonsilencing dsRNA group × 100. Results were analyzed statistically.

### Statistical analysis

Data are presented as the means ± standard deviation. Statistical analysis was performed using SPSS 15.0 software (SPSS, Inc., Chicago, IL, USA). P<0.05 was considered to indicate a statistically significant difference.

## Results

### Efficacy of siRNA expression vectors in transfection

**Figure 1:**
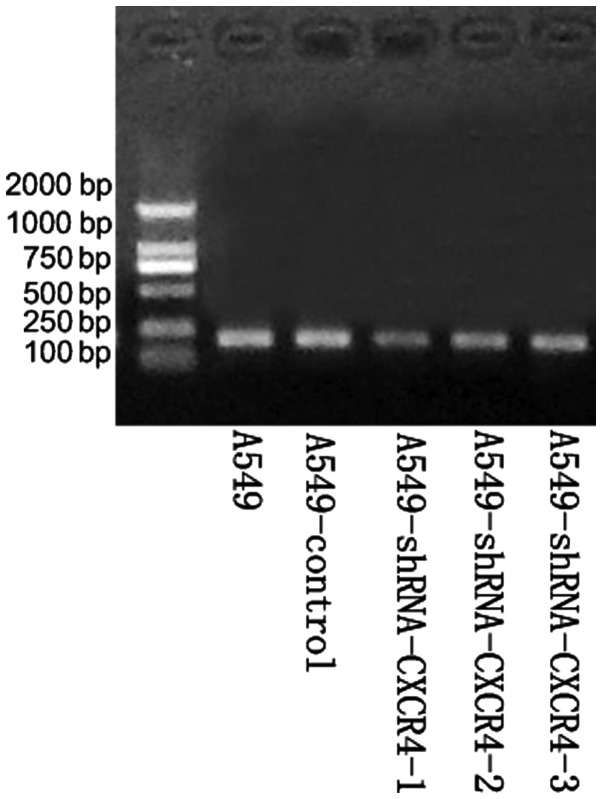
Identification of the tandem recombinant plasmid pBSilence1.1-CXCR4-1, -2 and -3 digested by restrictive enzyme. Fragments were 900 bp in length following digestion by *Sac*I. CXCR4, CXC chemokine receptor 4; shRNA, short hairpin RNA.

**Figure 2:**
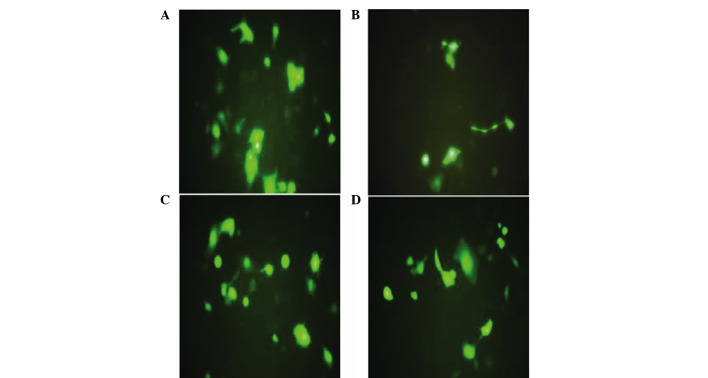
Plasmid transfection efficiency at 48 h following transfection. Expression of green fluorescent protein detected under a fluorescence microscope in (A) pBSilence1.1-CXCR4-1, (B) pBSilence1.1-CXCR4-2 and (C) pBSilence1.1-CXCR4-3 and (D) empty vector groups (magnification, ×200). CXCR4, CXC chemokine receptor 4.

Following PCR, recombinants were digested with the *Sac*I restriction enzyme. All plasmids, CXCR4-1, -2 and -3, produced ~900-bp DNA fragments, indicating that the target fragment had been successfully inserted into the pBSilence1.1 plasmid and in the right direction. DNA sequencing analysis confirmed that the sequence was consistent with the theoretical sequence (Fig. 1). In addition, restriction enzyme digestion and sequencing analysis confirmed that the recombinant vector, expressing three siRNA targeting the A549 CXCR4 gene in tandem, was constructed successfully. Following 48 h of transfection, no green fluorescence was identified in the untransfected group. By contrast, the expression of green fluorescent protein was detected under a fluorescence microscope in the empty vector and pBSilence1.1-CXCR4-1,-2 and -3 groups (Fig. 2). Transfection efficiency was determined as ~85%.

### siRNA-expressing vector inhibits CXCR4 mRNA expression

**Figure 3:**
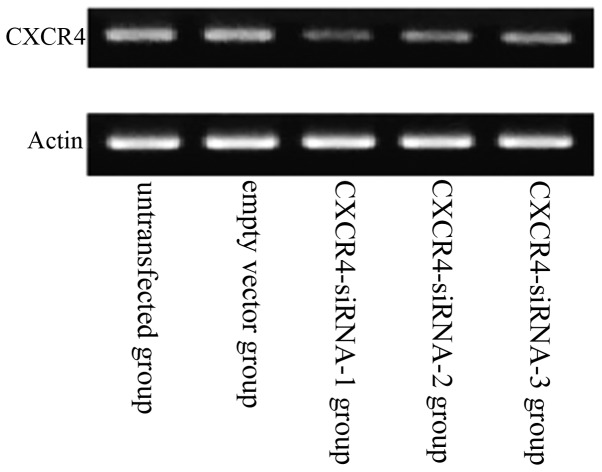
mRNA levels in A549 cells treated with siRNA-expressing vectors were assessed by real-time polymerase chain reaction analysis. CXCR4-siRNA-1, -2 and -3 inhibited CXCR4 mRNA at 48h following transfection of siRNA-expressing vectors in human lung cancer cells. Control cells were treated with empty vector and untransfected groups. siRNA, small interfering RNA; CXCR4, CXC chemokine receptor 4.

Compared with the untransfected group, the mRNA expression levels of CXCR4 in the A549 human lung carcinoma cell line were downregulated in the CXCR4-siRNA-transfected group (Fig. 3). Results also showed that siRNA targeting CXCR4-1, -2 and -3 decreased CXCR4 expression significantly at the mRNA level when compared with that of scrambled siRNA. Of the three CXCR4 siRNAs, the strongest interference efficiency siRNA was pBSilence1.1-CXCR4-1. However, CXCR4-1 exhibited the most significant inhibitory effects on the lung cancer cells (P<0.05). No significant difference was identified between the untransfected and empty vector groups (P>0.05).

### Effect of siRNA-expressing vectors on CXCR4 protein expression

**Figure 4:**
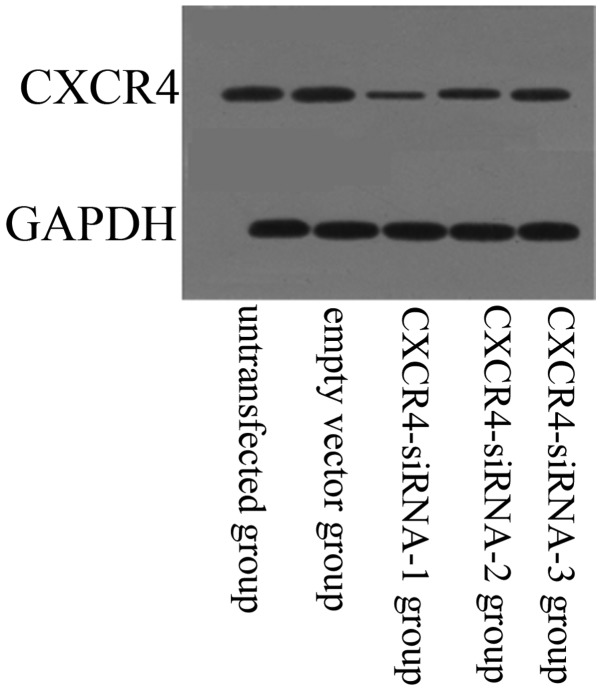
Effect of CXCR4-siRNA on CXCR4 protein expression in A549 cells. CXCR4, CXC chemokine receptor 4; si-RNA, small interfering-RNA.

The effect of siRNA-expressing vectors on target protein CXCR4 was examined by western blotting (Fig. 4). Compared with the untransfected group, protein expression levels of CXCR4 were downregulated in the CXCR4-siRNA transfected group (P<0.05). In addition, results showed that siRNA targeting CXCR4-1, -2 and -3 decreased CXCR4 expression significantly at the protein level when compared with that of scrambled siRNA. However, CXCR4-1 exhibited the most significant inhibitory effects on the lung cancer cells (P<0.05). No significant difference was identified between the untransfected and empty vector groups (P>0.05).

### Effect of siRNA-expressing vectors on cell proliferation

**Figure 5:**
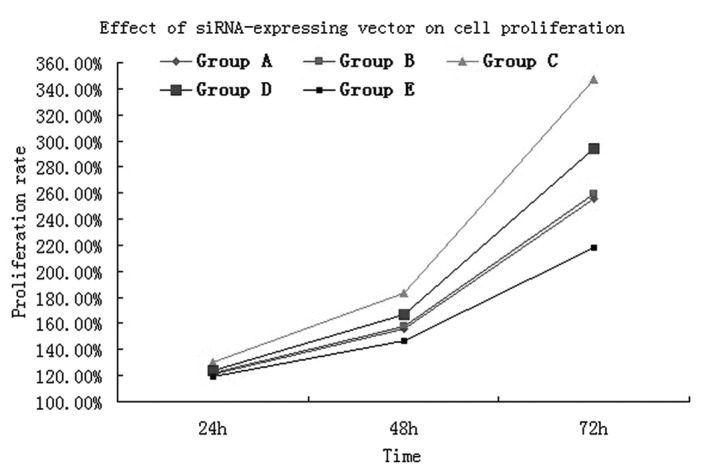
Effect of siRNA-expressing vectors on the cell proliferation of A549 cells following transfection. Cells were cultured in 96-well plates and cell viability was determined by MTT assay at 24, 48 and 72 h, respectively, following transfection. si-RNA, small interfering-RNA. Group A, normal A549 cells; group B, empty vector; group C, normal A549 cells and 100 ng/ml CXCL12; group D, CXCR4-siRNA A549 cells and 100 ng/ml CXCL12; and group E, CXCR4-siRNA A549 cells.

MTT assay results showed that following cultivation for 24 h, the cell proliferative activity in groups A (normal A549), B (empty vector), C (normal A549 and 100 ng/ml CXCL12), D (CXCR4-siRNA A549 and 100 ng/ml CXCL12) and E (CXCR4-siRNA A549) was 0.378±0.002, 0.380±0.002, 0.402±0.003, 0.385±0.002 and 0.373±0.002, respectively (Table I). Following A549 cell interference with CXCR4, the proliferative activity was significantly lower when compared with that of the normal A549 cells (t=12.57, P<0.05). While under the effect of chemokine CXCL12, the proliferative activity between RNAi-treated A549 cells and normal cells was evident and, when compared with normal A549 cells, a marked and statistically significant difference was identified (t=5.383, P<0.05). Cell proliferation was verified by MTT assay and for 48 and 72 h, the results were the same (Fig. 5). The analysis confirmed that the CXCL12/CXCR4 biological axis can induce lung cancer cell proliferation.

### Effect of siRNA-expressing vectors on cell invasion and migration

**Figure 6:**
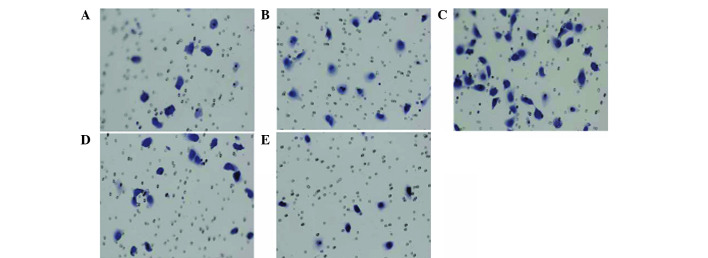
Effect of siRNA-expressing vectors on cell invasion and migration in each group (A, B, C, D and E). (A) A549 cells in DMEM culture medium without CXCL12. (B) A549 cells in DMEM culture medium containing 30 ng/ml CXCL12. (C) A549 cells in DMEM culture medium containing 100 ng/ml CXCL12. (D) RNA-interfered A549 cells in DMEM culture medium containing 100 ng/ml CXCL12. (E) RNA-interfered A549 cell inDMEM culture medium without CXCL12. siRNA, small interfering RNA; BSA, bovine serum albumin; DMEM, Dulbecco’s modified Eagle’s medium.

To further clarify the impact of the CXCL12/CXCR4 interaction on the migration capacity of lung cancer cells *in vitro*, chemotaxis and the chemotactic invasion response of lung cancer cells to the CXCR4 ligand, CXCL12, were detected following the downregulation of CXCR4 expression. The chemotactic invasion assay was performed using a Transwell chamber with CXCL12 as a chemoattractant, and the results showed that CXCL12 may induce various degrees of cell chemotactic invasion. In addition, the A549 cell line was able to spontaneously pass through the microporous membrane without CXCLl2 induction (Fig. 6) and the average transmembrane cell number was ~13.9±2.1 (Fig. 6A). Under the induction of 30 ng/ml CXCLl2, the number of A549 cells passing through the microporous membrane was higher than that of the control group, and the average transmembrane cell number was ~17.0±2.9 (Fig. 6B; t=2.988, P<0.05). When the concentration of CXCLl2 was increased to 100 ng/ml, the number of A549 cells passing through the microporous membrane increased significantly compared with that of the 30 ng/ml group and the average transmembrane cell number was ~30.6±7.3 (Fig. 6C; t=4.538, P<0.05). As demonstrated above, CXCLl2 may induce and enhance the chemotactic invasion ability of the CXCR4^+^ A549 cells and the chemotactic invasion ability enhanced gradually with the increase of CXCLl2 concentration in a concentration-dependent manner.

Following RNAi treatment, the number of A549 cells passing through the microporous membrane was 9.7±2.7 (Fig. 6E; t=3.953, P<0.05). While under the induction of 100 ng/ml CXCLl2, the number of RNA-interfered A549 cells passing through the microporous membrane was ~20.1±2.4 (Fig. 6D; t=5.568, P<0.05). Under the effect of 100 ng/ml CXCLl2, the number of A549 cells passing through the microporous membrane markedly decreased compared with that of the normal A549 group. Invasion analysis found that CXCL12 is mediated by CXCR4. If the expression of CXCR4 is effectively suppressed, the binding capacity between CXCL12 and CXCR4 is likely to be reduced and, thus, prevent CXCLl2 from exerting its biological function. If the expression of CXCR4 is suppressed, CXCLl2 is likely to lose specific binding sites even in the presence of CXCLl2 induction. Therefore, the chemotactic invasion capacity is not likely to increase, indicating that CXCLl2-induced chemotaxis or chemotactic invasion is mediated by CXCR4 specificity.

## Discussion

There is increasing evidence to suggest that the CXCL12/CXCR4 chemokine axis is important for the cell invasion and migration of several types of tumor, particularly lung cancer. It has been shown that a number of NSCLC cell lines express high levels of CXCR4, which is associated with aggressive behavior, and that CXCL12-activated CXCR4 promotes migration and invasion of these cell lines *in vitro* (11,13). Furthermore, preferential sites of lung cancer metastases *in vivo* exhibit significantly higher levels of CXCL12 protein expression compared with that of the primary tumor or plasma levels, indicating that a chemotactic gradient may be established between the site of the primary tumor and metastatic sites (14). The results of previous studies using various cancer cell lines have shown that inhibition of CXCR4 reduces the frequency of metastasis, indicating that the receptor is essential for tumor cell dissemination and invasion of tissues (15,16). In the present study, siRNA-mediated downregulation of CXCR4 expression in human lung cancer cells led to a significant decrease in A549 cell proliferation and invasion. This result is consistent with previous studies showing that CXCR4 mediates the invasive and metastatic potential of lung cancer cells (17). The direct effect of CXCL12/CXCR4 in tumor metastasis is that CXCL12 increases CXCR4-mediated motility, and the cell surface expression of integrins is mediated by the phosphorylation of extracellular signal regulated kinase (ERK) and downstream activation of the IKKαβ/NFκβ/RELA signaling (18). In addition, it has previously been reported that following binding to CXCR4, CXCL12 induces the mobilization of calcium, decreases the levels of cyclic AMP within cells and activates multiple signal transduction pathways, including PI3K/Akt/eNOS, which may enhance cell proliferation, migration, survival and angiogenesis signals by inducing eNOS activity (19).

RNAi is characterized by high efficiency, high specificity and low toxicity of post-transcriptional gene silencing, mediated by ds siRNAs. siRNA has become a powerful tool for studying gene function in carcinoma and viral disease therapy (15). Silencing is carried out by an RNA-induced silencing complex-associated RNase III-like endonuclease that cleaves the target homologous mRNA. The technology of RNA silencing is likely to have a major impact on the treatment of human diseases, particularly cancer (20,21). In the present study, three pairs of ds siRNA oligonucleotides were designed and constructed against CXCR4. These transcripts form a shRNA with an inverted repeat sequence separated by a short loop sequence. Three siRNAs targeting various sequences of human CXCR4 were cloned into a pBSilence1.1 vector for siRNA expression. The shRNA was processed into functional siRNA to degrade target mRNA and silence the expression. The cationic liposomal method has been widely used due to its ease, high transfection efficacy, widespread application and non-immunogenicity. Certain studies have achieved particularly high transfection efficiencies by using adenoviral vector-mediated siRNA delivery (22). Fluorescence microscopy was used to monitor the cell plating and transfection efficacy, which for A549 lung cancer cells was >85%. In addition, the suppressed expression of CXCR4 was confirmed by western blotting and RT-PCR at protein and mRNA levels, respectively. The results revealed that the RNAi constructs induced the selective degradation of CXCR4 mRNA and thereby decreased CXCR4 protein expression levels in lung cancer cells.

The proliferation of the A549 lung cancer cell line in response to CXCL12 was found to be reduced by the downregulation of CXCR4 expression by the pBSilence1.1-siRNA-CXCR4 vector, as determined by MTT assay. This led to the examination of the effects of CXCL12 stimulation on the A549 cell line. The results of the *in vitro* proliferation assay revealed that the reduction in cell absorbance in the CXCR4-siRNA group was greater compared with that of the untransfected and empty vector groups at 24, 48 and 72 h following transfection, respectively. This indicated that CXCR4 functions as a positive regulator in the growth of A549 cells and, thus, supports A549 cell proliferation. CXCL12 promoted the colony-forming capacity of A549 cells and CXCR4-positive cells were highly viable in response to CXCL12. By contrast, the proliferation of A549 cells was significantly reduced by CXCR4-siRNA, indicating that the downregulation of CXCR4 impaired the ability of the lung cancer cells to grow. The inhibitory effect was not time dependent, as no differences in CXCR4 inhibition were identified at 24, 48 and 72 h, respectively. This result indicated that the CXCL12/CXCR4 signaling pathway promotes tumor cell proliferation and is consistent with previous studies showing that the CXCL12-CXCR4 axis supports cancer cell growth (22). The mechanisms and signaling pathways involved in CXCL12/CXCR4 activation in NSCLC reported by Lee *et al* indicated that ERK activation is a key pathway in NSCLC development (23). Distant sites where CXCL12 is highly expressed may serve as favorable niches for metastasis to occur. The CXCL12/CXCR4 loop may stimulate tumor cell proliferation and induce extracellular matrix rearrangement, necessary for metastasis formation (24).

The current study also investigated the metastatic potential of lung cancer cell line A549 in response to CXCL12, which may be reduced by the downregulation of CXCR4 expression by the pBSilence1.1 vector, as determined by the Transwell assay. siRNA-CXCR4 mediated the downregulation of CXCR4 expression in human lung cancer cells and led to a significant decrease in the invasion and migration of A549 cells. By contrast, CXCR4-positive cells were highly invasive in response to CXCL12 and the CXCL12 mediated chemotaxis was dose dependent, indicating that the downregulation of CXCR4 had impaired the ability of the lung cancer cells to migrate. This result shows that CXCR4 mediates the invasive and metastatic potential of lung cancer cells, indicating that CXCR4 is important for the invasion and migration of lung cancer A549 cells toward CXCL12. Similarly, Sun *et al* demonstrated that the CXCR4-CXCL12 interaction and downstream signaling promoted the growth/survival of tumor cells, allowing them to grow in distant and less favorable sites (25).

In conclusion, the results of the current study indicate that CXCR4 siRNA treatment may significantly inhibit the growth, invasion and metastasis of lung cancer cells. Thus, we propose that CXCR4 may represent a therapeutic target for lung cancer patients, and that RNAi with siRNA targeting CXCR4 may establish an effective strategy for the treatment of lung cancer.

## Figures and Tables

**Table I:** Effect of siRNA expression vector on the cell proliferation of A549 cells following transfection.

Group	OD (24 h)	OD (48 h)	OD (72 h)
A	0.368±0.002	0.471±0.002	0.704±0.003
B	0.380±0.002^a^	0.476±0.002^a^	0.711±0.009^a^
C	0.402±0.003^b^	0.542±0.005^b^	0.928±0.009^b^
D	0.385±0.002^b^	0.502±0.007^b^	0.798±0.005^b^
E	0.373±0.002^a^	0.449±0.009^b^	0.610±0.011^b^

Data are presented as the mean ± SEM of five independent experiments.

a P>0.05 and

b P <0.05, vs. the untransfected group.

A, CXCR4-siRNA A549 group; B, empty vector group; C, normal A549 + 100 ng/ml CXCL12 group; D, CXCR4-siRNA A549 + 100 ng/ml CXCL12 group; E, normal A549 group. siRNA, small interfering RNA; OD, optical density.
